# Cortex level connectivity between ACT-R modules during EEG-based n-back task

**DOI:** 10.1007/s11571-024-10177-y

**Published:** 2024-10-21

**Authors:** Debashis Das Chakladar

**Affiliations:** Machine Learning Group, Luleå University of Technology, Luleå, Sweden

**Keywords:** Electroencephalography, Adaptive control of thought-rational, Granger causality, Multivariate transfer entropy

## Abstract

Finding the synchronization between Electroencephalography (EEG) and human cognition is an essential aspect of cognitive neuroscience. Adaptive Control of Thought-Rational (ACT-R) is a widely used cognitive architecture that defines the cognitive and perceptual operations of the human mind. This study combines the ACT-R and EEG-based cortex-level connectivity to highlight the relationship between ACT-R modules during the EEG-based *n*-back task (for validating working memory performance). Initially, the source localization method is performed on the EEG signal, and the mapping between ACT-R modules and corresponding brain scouts (on the cortex surface) is performed. Once the brain scouts are identified for ACT-R modules, then those scouts are called ACT-R scouts. The linear (Granger Causality: GC) and non-linear effective connectivity (Multivariate Transfer Entropy: MTE) methods are applied over the scouts’ time series data. From the GC and MTE analysis, for all *n*-back tasks, information flow is observed from the visual-to-imaginal ACT-R scout for storing the visual stimuli (i.e., input letter) in short-term memory. For 2 and 3-back tasks, causal flow exists from imaginal to retrieval ACT-R scout and vice-versa. Causal flow from procedural to the imaginal ACT-R scout is also observed for all workload levels to execute the set of productions. Identifying the relationship among ACT-R modules through scout-level connectivity in the cortical surface facilitates the effects of human cognition in terms of brain dynamics.

## Introduction

Adaptive Control of Thought-Rational (ACT-R) is the widely used cognitive architecture based on a rigorous theory of human cognition Anderson et al. (2008). In the ACT-R model, the input-to-output conversion is performed with the help of different modules such as visual, motor, working memory (imaginal), declarative memory (retrieval), procedural module, and goal module. The visual and aural modules (i.e., perceptual modules) scan, perceive, and encode the visual and auditory information from the external environment. The motor/manual module produces the output. The goal module holds the current control information to perform the task. The declarative module (DM) stores all the knowledge-based information. DM used the retrieval buffer, which is used during the matching of specific requests of another module. The imaginal module works as an intermediate memory that stores the perceptual information from the visual module Anderson (2009). The central module (i.e., procedural module) communicates with the other modules to execute a specific task by implementing a set of productions. The cortex-level activation can be measured through different physiological measures such as EEG, Functional magnetic resonance imaging (fMRI), and positron emission tomographic (PET) imaging. However, EEG with high temporal resolution became more popular to identify the cortex-level activation over time with a person’s cognitive behavior. In van Vugt (2014), authors illustrated the relationship between the ACT-R model and Electroencephalography (EEG) oscillation power. The author found that the working memory of ACT-R has been related to the parietal theta band oscillation of EEG. In an EEG-based *n*-back task, the participant has to remember the stimulus with a longer sequence with the increasing value of *n*, which occupies more cognitive resources Chakladar et al. (2024, 2022). Apart from cognitive tasks Chakladar et al. (2022), EEG can also be used in neuro-recommendation system Panda et al. (2024) and neuromarketing application Panda et al. (2024). Cognitive load can be measured during attentional-based emotional tasks Mishra et al. (2023); Prasad et al. (2023), whereas the machine learning model (i.e.; multi-layer perceptron-based regressor) is used to find the cognitive dynamics during emotional tasks Panda et al. (2020); Prasad et al. (2024). The EEG-based neurocognitive mechanism is used to find the changes in prosocial emotional behaviors of participants Tarai and Bit (2021); Tarai et al. (2022). Each ACT-R module can be mapped to specific brain sources to identify the underlying brain dynamics with respect to cognitive processing. Therefore, it is important to identify cortical sources for ACT-R modules. Source localization methods have been used to find sources from the cortical surface from scalp EEG signal Mosher et al. (1999).

The source localization was performed in two ways: Dipole source localization and distributed source imaging. In the first approach, a localized set of dipoles is selected based on the prior assumption, assuming that scalp potential is generated from those dipoles. However, as the dipole localization is totally based on prior assumptions, the source localization can be biased for the missing relevant dipoles Michel and He (2019). On the other hand, in Distributed source imaging (Minimum Norm Dale and Sereno (1993), Low-resolution electromagnetic tomography (LORETA) Pascual-Marqui et al. (1994), Standardized low-resolution brain electromagnetic tomography (sLORETA) Pascual-Marqui (2002)), a large number of dipoles are distributed in fixed positions over the entire source space and the strength of dipoles is estimated to measure the scalp potential. Due to the minimum localization error and low model complexity Jatoi et al. (2014), the sLORETA-based source estimation method is used. sLORETA is widely used in EEG-based motor imagery application Li et al. (2019), stimuli-based Event-Related Potential (ERP) analysis Tsolaki et al. (2017). An extended version of the spatiotemporal source imaging method has been implemented to identify the brain network Sohrabpour et al. (2020). Their brain network estimated spatially coherent regions and temporally transformed information between them.

Three brain connectivity systems exist to communicate between different brain regions: structural, functional, and effective connectivity He et al. (2019). The synaptic connections between different brain regions represent structural connectivity. On the other hand, the functional connectivity system identifies the statistical relationship between anatomically separated brain regions. The causal activation between two brain regions is established by effective connectivity. In Sakkalis (2011), authors discussed functional connectivity analysis based on model-based techniques (correlation, magnitude squared coherence) and data-driven techniques (mutual information: MI, principal component analysis). They have also discussed effective connectivity methods such as Dynamic Causal Modeling: DCM, Granger causality: GC, and Partial directed coherence. In Chakladar et al. (2021), authors have developed a graphical brain network using GC and MI to measure the brain dynamics during the mental arithmetic task. A hybrid framework of Magnetoencephalography (MEG)/EEG-based GC analysis and source imaging technique is used to find the underlying brain networks in epilepsy application Sohrabpour et al. (2016). Protopapa *et al.*Protopapa et al. (2014) employed EEG-based GC analysis to reveal distinct network structures in various spatial working memory tasks. Epstein *et al.*Epstein et al. (2014) effectively identified epileptic seizure origins using GC and source localization of EEG. However, due to the non-linear characteristics of EEG, GC is not a good choice for identifying information flow between brain regions Vicente et al. (2011). Like GC, Transfer Entropy (TE) does not depend on the underlying model or prior information; rather, it assesses dynamic directional information flow between time series data in a non-linear manner Gao et al. (2018); Schreiber (2000). TE is an extension of mutual information that measures the directed information transfer between the time series of a source variable and a target variable Schreiber (2000). TE is also widely used to determine stimulus–response relationships and underlying brain dynamics Vicente et al. (2011). Information transfer between brain regions during sustained attention-driven driving has been measured using TE Huang et al. (2015). At the intermediate level of vigilance during driving, they found a strong association between frontal, parietal, and central brain regions. However, the bivariate analysis (i.e., bivariate transfer entropy: BTE/ TE) may infer spurious or redundant interactions where multiple sources provide the same information about the target Wollstadt et al. (2018). To overcome the issue of BTE, multivariate transfer entropy (MTE) has been implemented Bonmati et al. (2018). In MTE, multiple sources jointly transfer more information to the target than the contributions of an individual source Wollstadt et al. (2018); Novelli et al. (2019). MTE can model better Non-linear causal interaction between different brain regions than BTE and GC in EEG-based schizophrenia data analysis Harmah et al. (2020). The authors found a strong activation in the temporal lobe for schizophrenia patients with MTE. MTE model is also used for EEG-based face perception task Chakladar and Pal (2024).

**Fig. 1:**
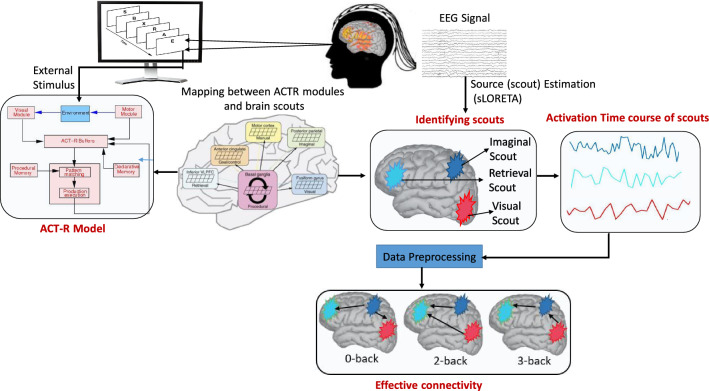
The proposed framework of scout-level connectivity between ACT-R modules. The scouts corresponding to the ACT-R modules are estimated from the EEG-source localization method (sLORETA), which is validated by the location of the Brodmann area. The connectivity between scouts is performed based on the activation time course of each scout. Effective (Granger causality, Multivariate transfer entropy) connectivity analyses are performed to identify the causal connectivity and information flow between the brain scouts

Most of the existing studies van Vugt (2014); Prezenski and Russwinkel (2016) discussed the relationship between ACT-R and scalp potential of EEG signal or highlighted the EEG-based source localization techniques Sohrabpour et al. (2016, 2020). However, the connectivity between ACT-R modules is also not explored. Therefore, this paper proposes a hybrid framework combining the ACT-R model and EEG-based source imaging technique. The proposed framework identifies the mapping between ACT-R modules and brain scouts (in the cortical surface) and then finds the connectivity among those scouts. The proposed framework is shown in Fig. 1. In the proposed model, I identify the brain scouts/sources for ACT-R modules using the sLORETA method and find the effective connectivity approaches (GC and MTE) between the time series of brain scouts. The novelties of the studies are as follows:

- This is the first work that combines the ACT-R model and EEG source imaging technique. The source imaging technique identifies the cortical sources/scouts related to the ACT-R modules, leading to the effects of neural activity on human cognition.
- To identify the causal effects and information flow among cortical sources, the proposed framework implements two widely used effective connectivity methods (GC and MTE). The statistical test also validates the direction of causal connectivity between sources.

The remainder of the paper is organized as follows. The proposed model is discussed in Sect. "Methods". The experimental results of the proposed model are represented in Sect. "Results". Finally, Sect. "Discussion and conslusion" highlights the discussion of the work and concludes the paper.

## Methods

This section is divided into two subsections: (a) EEG scouts estimation, and (b) Effective connectivity estimation between scouts. A detailed description of each subsection is mentioned below.

### EEG scouts estimation

The distributed source imaging method is established to find active localized sources/scouts with minimum localization error Jatoi et al. (2014). Among many distributed imaging methods, LORETA and sLORETA are mostly used in EEG-based cognitive and behavioral science applications Jatoi et al. (2014). LORETA introduces the minimization of the Laplacian of the sources, leading to a smooth (low resolution) distribution of the 3D activity Pascual-Marqui et al. (1994). LORETA assumes that the current density at any given point on the cortex is maximally similar to the average current density of its neighbor Jatoi et al. (2014). An advanced version of LORETA is sLORETA, where the current density is measured from the variance of the EEG noise and biological variance of the signal Pascual-Marqui (2002). The biological variance is assumed to be distributed uniformly across the brain, leading to zero localization error Jatoi et al. (2014). As the zero localization error efficiently estimates better sources, this paper used the sLORETA algorithm to estimate brain scouts at the cortex surface. Only visual stimuli (letters/digits in the *n*-back task) are used in the experimental dataset, and no auditory stimuli are used. The visual module of ACT-R is associated with the fusiform Gyrus (Brodmann’s area: BA 37), and the motor module is related to the primary motor cortex (BA 2 and 4) Qin et al. (2007) of the brain. The retrieval module of ACT-R is mapped with the dorsolateral prefrontal cortex (DLPFC). The DLPFC region is mapped to the BA 45 and 46 Qin et al. (2007). The working memory (imaginal) module is associated with a subregion of the parietal cortex on the border of the intraparietal sulcus (BA 7,39 and 40) Qin et al. (2007). The goal module is associated with the anterior cingulate cortex (BA 24, 32), and the procedural module with the basal ganglia Anderson et al. (2008). Three components of basal ganglia (striatum, pallidum, and thalamus) are used for controlling the input and output information of the brain and performing information flow among brain regions Houk and Wise (1995). The thalamus is mainly used for information flow and execution of instructions Sherman and Guillery (2002); therefore, I select it for mapping the production module of ACT-R.

### Effective connectivity estimation between scouts

**Fig. 2:**
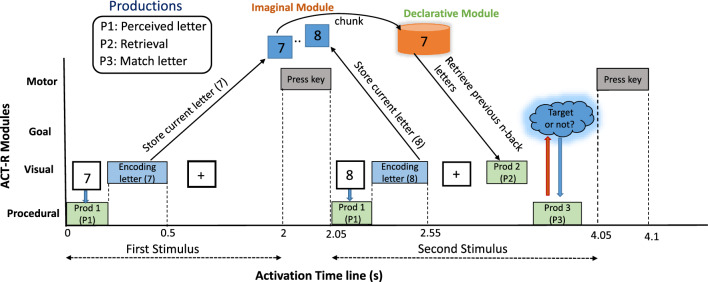
Activation time sequence diagram of ACT-R modules for EEG-based *n*-back task. The duration of each digit presentation (with fixation cross) is 2 s. Here, only the first two input digits are considered to illustrate the retrieval operation. For the first stimulus in the trial, the participant presses the button without checking the *target* letter. Activation time denotes the duration of the ACT-R module’s activation while the subject performs a specific event (i.e., digit encoding, pressing key, etc). After a digit presentation, a fixation cross appears in the rest state

After identifying the scouts of each ACT-R module (in the earlier section), the scouts’ time series is identified for the 4 s (display of two digits as per Fig. 2). The effective connectivity approaches are performed on the scouts’ time-series data. Here, two well-known effective connectivity methods (GC and MTE) are illustrated for identifying the information flow among brain scouts. GC is widely used for estimating brain connectivity in EEG-based cognitive workload studies Chakladar et al. (2021). However, due to the non-linear characteristics of EEG, a non-linear model-free method (MTE) is used to find the information flow among brain scouts. A detailed discussion of GC and MTE is defined below.

#### Granger causality

Granger causality (GC) predicts a one-time series of data from the past values of another time-series data. The GC (1) is mainly calculated by univariate and bivariate autoregressive (AR) models using variance (*Var*) of both the AR models’ residuals ($$e_x, e_{xy})$$ Cohen (2014). GC can be interpreted as if signal *Y* is causal to signal *X*, then *X* can be better predicted by incorporating the past values of *Y* than only information from itself. GC for *Y* to *X* is calculated using the following equation Cohen (2014).

1 $$\begin{aligned} GC(Y\rightarrow X)=ln\Bigg (\frac{Var(e_x)}{Var(e_{xy})}\Bigg ) \end{aligned}$$

#### Multivariate transfer entropy

Transfer Entropy (TE) is derived from the mutual information theory to find the conditional transitional probabilities between two paired processes. For two-time series data $X=x_t$ and $Y=y_t$, the delay embedded vector of *X* is defined by: $x_t^d=(x_t, x_{t-\tau },.., x_{t-(d-1)\tau })$; similar representation can also be done for $y_t^d$. The dimension of embedding space and delay are *d* and $\tau$, respectively. The entropy rate of the system *X* is the average number of bits that are required to represent an additional state, provided all previous states are known. The entropy rate for the time series *X* is defined below Huang et al. (2015):

2 $$\begin{aligned} h(x_{t+u}\vert x_t^d) =- \sum _{x_{t+u},x_t^d} p(x_{t+u},x_t^d)\log p(x_{t+u}\vert x_t^d) \end{aligned}$$

where, $p(x_{t+u}\vert x_t^d)=p(x_{t+u}, x_t^d)/ p(x_t^d)$. $p(x_{t+1}\vert x_t^d)$ denotes the transition probability (based on Markov process of order *d*). The prediction time is *u*, and $p(*)$ is the probability. If $p(x_{t+u}\vert x_t^d)=p(x_{t+u}\vert x_t^d, y_t^m)$, then no information transfer takes place between *X* and *Y*. The amount of information transfer from process *X* to *Y* can be defined by Transfer Entropy ($TE(X \rightarrow Y$)) Schreiber (2000), which is calculated as follows:

3 $$\begin{aligned} TE (X \rightarrow Y)= \sum p(y_{t+u},y_t^d,x_t^m )\log \Big (\frac{p(y_{t+u}\vert y_t^d,x_t^m )}{p(y_{t+u}\vert y_t^d)}\Big ) \end{aligned}$$

where, $x_t^m={\{x_t, x_{t-\tau },...,x_{t-(m-1)\tau }}\}$ describing the time series *Y* depends on *m* states of *X*.

Now, let’s consider the $X_n$, $Y_n$, and $Z_n$ as the stochastic variables obtained after sampling the processes at present *n*. The vector variables of past processes of *X*, *Y* and *Z* are denoted as $X_n^-=[X_{n-1}, X_{n-2},....]$, $Y_n^-=[Y_{n-1}, Y_{n-2},....]$ and $Z_n^-=[Z_{n-1}, Z_{n-2},....]$ respectively. Then, the MTE from *X* to *Y* conditioned on *Z* is defined below Montalto et al. (2014):

4 $$\begin{aligned} MTE(X \rightarrow Y \vert Z)&=\sum p(Y_n, Y_n^-, X_n^-, Z_n^-) \nonumber \\&\quad \log \Big (\frac{p(Y_n \vert Y_n^-,X_n^-,Z_n^-)}{p(Y_n \vert Y_n^-,Z_n^-)}\Big ) \end{aligned}$$

Here, the MTE is calculated using the IDTxl tool Wollstadt et al. (2018).

## Results

The results section is divided into four subsections, namely (a) Dataset and experimental analysis, (b) Sequential activities of ACT-R modules & Cortex activation analysis, (c) Effective connectivity analysis among brain scouts, and (d) Statistical analysis of Granger prediction results. A detailed discussion of each section is mentioned below.

### Dataset and experimental analysis

An open access dataset Shin et al. (2018) is used to evaluate the proposed model. Therefore, no ethical permission is required for data collection. The dataset contains EEG recordings of twenty-six subjects (9 males and 17 females, average age of 26.1±3.5 years). EEG data were recorded using 30 EEG electrodes according to the international 10-5 electrode placement system (Fp1, Fp2, AFF5h, AFF6h, AFz, F1, F2, FC1, FC2, FC5, FC6, Cz, C3, C4, T7, T8, CP1, CP2, CP5, CP6, Pz, P3, P4, P7, P8, POz, O1, O2, TP9 (reference) and TP10 (ground)). The sampling frequency was 200 Hz. The raw EEG was already filtered (fourth-order of Chebyshev type II) with a passband of $1-40$ Hz to remove high-frequency noise from the EEG. The fourth-order Chebyshev type II filter provides a sharp transition between the passband and stopband. This characteristic of the Chebyshev type II filter effectively isolates the desired frequency components of the EEG signal from noise, especially when the noise is close to the frequency band of interest. Moreover, the *stopband attenuation* feature of this filter is useful for filtering out specific noise components, leading to better filtered EEG data Sree et al. (2023).

In the *n*-back test, participants need to identify the letter/digit presented *n* stimuli earlier in the sequence. The experimental dataset includes three sessions, each with three series of 0, 2, and 3-back (i.e.; $n=0,2,3$) tasks arranged in a counterbalanced order. Participants completed nine series of *n*-back tasks in total. Each series consisted of a 2s instruction, a 40s task period, and a 20s rest period. During the rest period, a fixation cross was shown. In the task period, a random digit/number appeared in every 2*s*, with 20 trials per series where the *targets* appeared with a 30% chance (70% *non-targets*). Each number was displayed for 0.5s, followed by a fixation cross for the remaining 1.5s. For the 0-back task, participants either pressed the *target* or *non-target* button without recalling any earlier digit. For the 2- and 3-back tasks, they pressed the *target* button if the current number matched the one 2 or 3 positions back, respectively. Participants completed a total of 180 trials (20 trials $\times$ 3 series $\times$ 3 sessions) for each *n*-back task across the three sessions. For computational constraints, randomly two consecutive digits of the task are selected to identify the activation time sequences of different ACT-R modules.

**Fig. 3:**
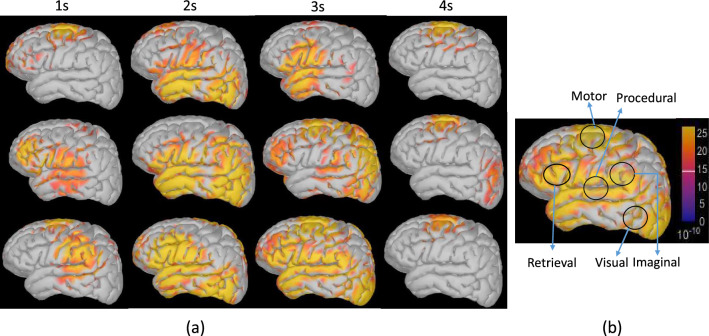
**a** Time-wise activation of brain scouts for different workload levels. Top row: 0-back, middle row: 2-back, and last row: 3-back task. The activation time is written at the top of each cortex image. **b** The scout region in each ACT-R module is marked in a circle. Bright (yellow) and dark (blue) colors represent the high and low brain activations respectively

### Sequential activities of ACT-R modules & cortex activation analysis

All the ACT-R modules are activated at a specific time in the *n*-back task (from visualizing the input stimuli to pressing the key). ACT-R modules are communicated using their buffer. The activation sequence diagram of ACT-R modules according to the experimental dataset is shown in Fig. 2. In each trial of the *n*-back dataset, a digit is presented for 2 s (0.5 s for the input letter and 1.5 s for the fixation cross). The diagram shows the activities of different ACT-R modules according to the timings of the two input letters of a single trial. The goal module only holds the current control information for the task, which is checked with the execution of the final production (P3). Therefore, the execution of the goal module occurs almost simultaneously with the P3 (within the same period of P3), so the goal module is excluded from the experimental analysis. The first input letter (7) appears on the computer screen in the sequence diagram, and the first production (P1) is fired to perceive the letter. Next, the visual module encodes that input letter, and after a slight delay, that letter is stored in the imaginal module. The imaginal module works as an intermediate or short-term memory in the ACT-R module. After the presentation of input stimuli, a fixation cross appears for 1.5 s, and at 2 s, the subject presses the key based on target or non-target stimuli. The next input (8) appears on the screen and follows a procedure similar to the first input. By this time, the imaginal module transfers the previous input letter (7) as a chunk to the declarative module. Once the second input letter is encoded & stored, the second production (P2) is fired to fetch the previous letter from the declarative memory. The Goal module holds the present input stimuli, and when the third production (P3) is fired, the Goal module communicates with the procedural module to check whether the present input is the same as the previous letter in the sequence (retrieved from the declarative memory). Finally, based on the matching or non-matching letter of current stimuli with the previous one, the subject presses the key by calling the motor module of ACT-R.

After identifying the ACT-R activation time sequence (refer to Fig. 2), I identify the activation of brain sources for each ACT-R module. The mapping of ACT-R modules and its corresponding brain sources/scouts are identified based on anatomical positions of BA Qin et al. (2007) (refer to Fig. 3b). Initially, the brain scouts for each *n*-back task are identified using the *sLORETA* method. To illustrate the ACT-R modules’ operations, I used two consecutive letters in a trial. Execution of other letters follows the same process. The activation of scouts in the cortex surface for the different time durations (interval of 1 s) is presented in Fig. 3. As neural activation is distributed over the cortex surface, scouts and their nearest locations are activated during a specific time. The cortex activation for 0, 2, and 3-back tasks is presented at the top, middle, and bottom rows of Fig. 3a. I start the scout activation after presenting input stimuli (i.e., 1 s). At 1 s, procedural (basal ganglia), visual (fusiform gyrus: BA-37), and imaginal modules are activated to present and process the visual stimulus of the 0-back task. Production 1 (P1) is activated for perceiving the input letter (refer to Fig. 2). At 2 s, the first letter presentation is completed, and the subject presses the button, so the motor module (BA-2,4) is activated. After 2 s, a second letter appears on the screen. From 2 to 3 s, multiple works perform in a sequence: (a) visual perception of the second letter and firing of P1 for processing the letter, (b) storing the second letter in the imaginal module, (c) fetching the previous letter from the declarative/retrieval module (BA-45) after execution of P2, and (d) P3 is fired to find a match between the retrieved letter and current letter. The goal module is communicated with P3 to find the matched or non-matched letter. Finally, at 4 s, the motor module is activated while the subject presses the button.

### Effective connectivity analysis among brain scouts

**Fig. 4:**
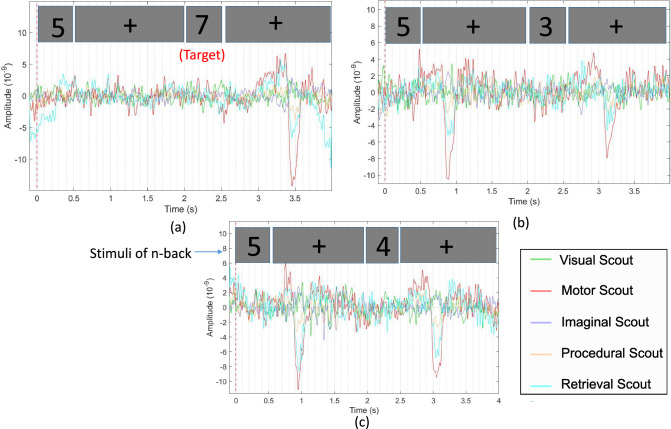
Scouts’ time series of n-back task: **a** 0-back, **b** 2-back, and **c** 3-back. Each stimuli time duration is 2 s (0.5 s for digit and 1.5 s for fixation cross.)

After the scout identification, the time series of each scout corresponding to the ACT-R module is extracted. The time series are extracted for target and non-target stimulus for all workload levels (0,2 and 3-back). Then, the band-pass filter (0-32 Hz) is applied to remove noise from time series data. For simplicity, the scouts’ time series of target stimuli is presented in Fig. 4. Once the time-series extraction is performed, the effective connectivity analysis among scouts is identified through GC and MTE. The activation time series of ACT-R scouts is extracted using the brainstorm software Tadel et al. (2011). The information flow among ACT-R modules through GC and MTE methods is presented in the subsequent subsections.

#### Information flow analysis using GC

**Fig. 5:**
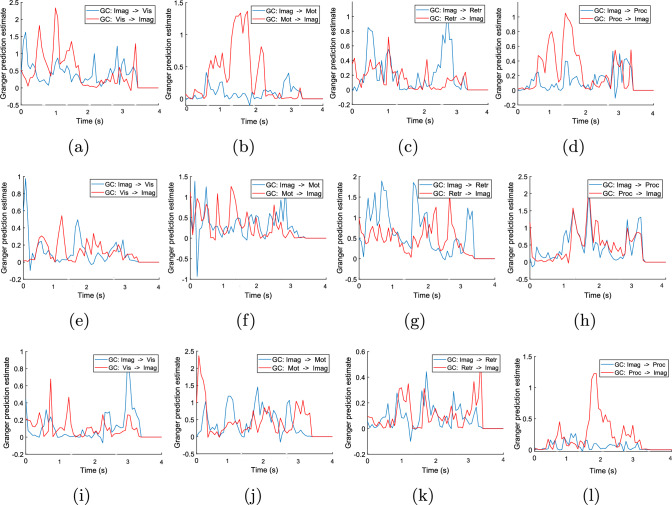
Effective connectivity (GC) analysis among activation time series of ACT-R scouts for *target* stimuli (**a**–**l**). GC analysis is divided into 0-back (**a**–**d**), 2-back (**e**–**h**), and 3-back (**i**–**l**) tasks. The time segment for computing Granger prediction is 2ms. The order of the BVAR model is 7. Abbreviations in legends of images are as follows: imaginal (Imag), retrieval (Retr), motor (Mot), procedural (Proc) and visual (Vis)

**Fig. 6:**
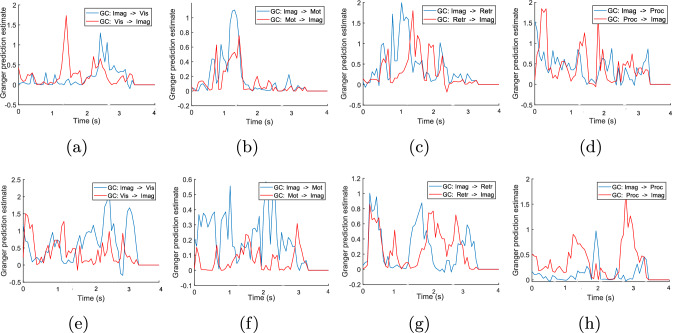
Effective connectivity (GC) analysis among ACT-R scouts for *non-target* stimuli. GC plots are divided into two workload states 2-back(**a**–**d**) and 3-back(**e**–**h**). The parameters (time segment of GC prediction and BVAR model order) remain the same as Fig. 5

The GC analysis between two scouts is estimated using bi-variate autoregressive models (BVAR) and time windows (i.e., the segment of the scout’s activation data). The BVAR model’s order is selected using the Bayesian information criterion using the Source Information Flow Toolbox (SIFT) Delorme et al. (2011). From SIFT, the optimal model’s order for the experiment is set as seven. The GC is computed based on the time series data of scouts. As transient connectivity is lost with a large time window, a shorter time window (i.e., 2 ms) is used to estimate the GC efficiently Cohen (2014). Moreover, using a short time window in GC analysis offers several significant advantages, particularly in enhancing temporal resolution. First, it allows for the detection of rapid and transient interactions between variables that may be missed with longer windows. This higher temporal granularity enables researchers to capture detailed dynamics and finer-scale causal relationships Cohen (2014). The GC is calculated for this time segment sequentially for the entire scout time series data of 4 s. Finally, the GC analysis for all such time segments is merged to find the final effective connectivity between the two scouts. The result of GC between different scouts across all the workload levels is presented in Fig. 5a–l. The direction of GC between two scouts (A$\rightarrow$B) also refers to the information flow between those scouts. The direction of information flow using GC is also validated through the statistical analysis (refer to Table 2). Here, the GC analysis are performed for target (Fig. 5) and non-target stimuli (Fig. 6) of 2-back and 3-back tasks (as 0-back task only includes fixation cross). The Imaginal module of ACT-R works as a working memory that stores the information and is manipulated during problem-solving Peebles (2019), so I used the GC analysis from Imaginal to other ACT-R modules and vice-versa. The main aim of the paper is to check the information flow between different ACT-R modules (e.g.; scouts) during different events of n-back tasks such as: the presentation of stimuli of n-back task (imaginal-visual modules, vice-versa), information processing/recall (imaginal-procedural/retrieval, vice-versa), and button press event (imaginal-motor, vice-versa). So I used the GC analysis from Imaginal to other modules. In the 0-back task, visual to-imaginal ACT-R scout activation is observed during stimulus presentation ($0.5-1$ s) (Fig. 5a). Information flow from procedural to imaginal ACT-R scout is observed during stimulus presentation (Fig. 5d). For the target stimuli of 2 and 3-back tasks, a high peak in GC value is observed between visual and imaginal ACT-R scout immediately after the appearance of visual stimuli (Fig. 5e and i). A larger GC value leads to better information flow between brain scouts. For all the 2 and 3-back tasks, information flow is observed from the procedural to the imaginal ACT-R scout (Fig. 5h and l) for the execution of a set of productions (P1, P2, and P3 in Fig. 2). For the execution of P2 and P3, there is an information flow in both directions between the imaginal and retrieval ACT-R scouts for storing the current letter into short-term memory (imaginal buffer) and recalling past *n* letters from short-term memory (Fig. 5g and k). For all *n*-back tasks, causal effects are observed from the motor to imaginal ACT-R scout (Fig. 5f and j) at 2 s, when the subject presses the button. In the case of non-target stimuli, I found similar GC flow in visual and imaginal ACT-R scouts (Fig. 6a and e). Information flow between the imaginal and retrieval ACT-R scouts and vice-versa is also observed to check whether the current letter is *target* or not (Fig. 6c and g). Information flow from procedural to the imaginal ACT-R scout is observed for 2-back and 3-back tasks for executing the productions (Fig. 6d and h).

#### Information flow analysis using MTE

**Fig. 7:**
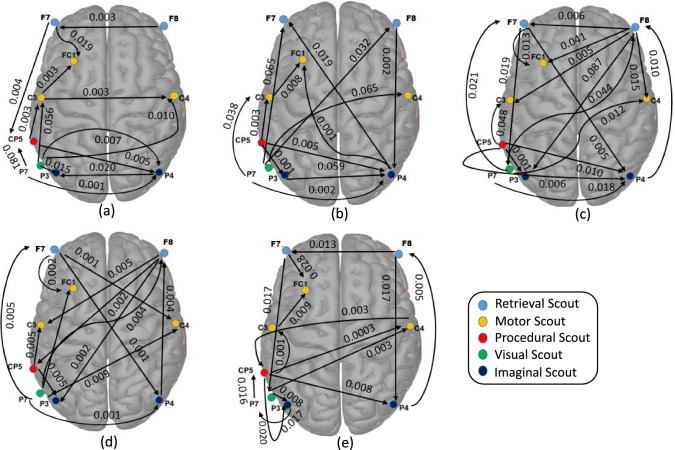
Non-linear causal connectivity analysis using MTE based on channels mentioned in Table 1: **a** 0-back, **b** 2-back (target), **c** 2-back (non-target), **d** 3-back (target), and **e** 3-back (non-target). Different colored EEG channels denote different ACT-R modules. The edges represent the MTE values between the two channels

The non-linear effective connectivity analysis is performed using MTE. The MTE computation is performed using the IDTxl tool Wollstadt et al. (2018), where the transfer entropy is depicted between a set of significant sources and the target. Here, the MTE connectivity is performed through the time series of scalp electrodes. The MTE connectivity analysis is performed using the “IDTxl" tool Wollstadt et al. (2018), where the input dimension is no of process/channels and no of samples. Therefore, the scalp electrodes for scouts are required for MTE analysis. The scalp electrodes for each ACT-R module are selected in such a way so that they are validated through BA’s/nearest BA’s anatomical location Qin et al. (2007). The ACT-R module and EEG electrode with the nearest BA location are shown in Table 1.^1^ The results of MTE connectivity graphs among different ACT-R modules are shown in Fig. 7. All MTE graphs are demonstrated based on the activation time of the presentation of two stimuli (i.e., 4 s).

For all types of *n*-back tasks, it can be observed that the visual ACT-R module is mostly activated for stimulus presentation (digit and fixation cross). For the 0-back task (Fig. 7a), the information flow is transferred from visual (EEG channels: P7) to imaginal (EEG channels: P3, P4), procedural (EEG channels: CP5), and motor (EEG channels: C3, C4) ACT-R scouts. For the 2 and 3-back tasks (Fig. 7b–e), information flow is observed from visual (EEG channels: P7) to imaginal (EEG channels: P3/P4), retrieval (EEG channels: F7/F8), and motor (EEG channels: C3/C4) ACT-R scouts. Similar to GC, information flow from visual-to-imaginal ACT-R scouts exists for storing the current stimuli in short-term memory (e.g., imaginal ACT-R module). On the other hand, the connectivity between the imaginal (EEG channels: P4) and retrieval (EEG channels: F8) ACT-R scouts is present for 2 and 3-back tasks for retrieving previous digits from memory. Procedural (EEG channels: CP5) to imaginal (EEG channels: P3) ACT-R scout connectivity is observed for 2 and 3-back tasks during the execution of productions P1, P2, and P3. GC analysis also demonstrates similar findings. However, GC requires the AR model in its backend to execute, whereas MTE does not need any execution model.

**Table 1:** Mapping between ACT-R module, EEG electrodes, and Brodmann area (BA)

ACT-R modules	EEG electrodes (BA)
Imaginal	P3, P4(39), Pz(7)
Retrieval	F7, F8(47)
Visual	P7(37)
Motor	C3,C4(1),Cz(5),FC1,FC2(6)
Procedural	CP5, CP6 (40)

### Statistical analysis of granger prediction results

As the Granger prediction depicts the ratio of error variances of two-time series data (predicted from the autoregressive model), the statistical significance between two-time series can be checked using the *F*-test. For all workload levels, the *F*-test (Table 2) is performed to find the direction of causality between brain scouts with the significance level of $p=0.05$. The analysis is performed between the activation time series obtained from brain scouts of each ACT-R module. The following hypothesis needs to be established to find whether the Scout 1 granger causes Scout 2.

$H_0$ *: Error Variance (Scout 2) = Error Variance (Scout 1)*

$H_a$*: Error Variance (Scout 2)* > *Error Variance (Scout 1)*

If the error variance of two scouts’ time series is the same (i.e., $H_0$), then there is no Granger Causality (GC) from Scout 1 to Scout 2. On the other hand, if the error variance of the scout 1-time series is lesser than scout 2, then scout 1 Granger causes scout 2 ($H_a$). $H_0$ is rejected, if the *p* value of Granger prediction (scout 1$\rightarrow$ scout 2) < 0.05 (at 5% level of significance). Here, five ACT-R modules are considered, such as scout 1: imaginal, scout 2: visual, scout 3: motor, scout 4: retrieval, and scout 5: procedural in the experiment and perform the *F*-test to identify the direction of Granger prediction. The prediction validates the direction of effective connectivity between two brain scouts (in terms of ACT-R modules).

**Table 2:** Statistical result of Granger prediction using *F*-test.

Workload level	S1$\rightarrow$S2	S1$\rightarrow$S3	S1$\rightarrow$S4	S1$\rightarrow$S5
0-back	*p*=0.1102, *p*=**0**.**0146** (S2$\rightarrow$S1)	*p*=0.1725, *p*=0.6202 (S3$\rightarrow$S1)	*p*=0.3887, *p*=0.8490 (S4$\rightarrow$S1)	*p*=0.1256, *p*=**0**.**0035** (S5$\rightarrow$S1)
2-back (target)	*p*=0.7220, *p*=**0**.**0141** (S2$\rightarrow$S1)	*p*=0.8789, *p*=0.7570 (S3$\rightarrow$S1)	*p*=**0**.**0178**, *p*=**0**.**0362** (S4$\rightarrow$S1)	*p*=0.4743, *p*=**0**.**0340** (S5$\rightarrow$S1)
3-back (target)	*p*=0.3518, *p*=**0**.**0366** (S2$\rightarrow$S1)	*p*=0.8570, *p*=0.9420 (S3$\rightarrow$S1)	*p*=**0**.**0407**, *p*=**0**.**0315** (S4$\rightarrow$S1)	*p*=0.9402, *p*=**0**.**0422** (S5$\rightarrow$S1)
2-back (non-target)	*p*=0.2943, *p*=**0**.**0468** (S2$\rightarrow$S1)	*p*=0.8205, *p*=0.1398 (S3$\rightarrow$S1)	*p*=**0**.**0420**, *p*=**0**.**0131** (S4$\rightarrow$S1)	*p*=0.3993, *p*=**0**.**0344** (S5$\rightarrow$S1)
3-back (non-target)	*p*=0.1293, *p*=**0**.**0105** (S2$\rightarrow$S1)	*p*=0.5758, *p*=0.0789 (S3$\rightarrow$S1)	*p*=**0**.**0248**, *p*=**0**.**0487** (S4$\rightarrow$S1)	*p*=0.3013, *p*=**0**.**0298** (S5$\rightarrow$S1)

Scout 1: imaginal, scout 2: visual, scout 3: motor, scout 4: retrieval, scout 5: procedural. In table entries, scouts are abbreviated as *S*. GC causes from scout $x \rightarrow$ scout *y* when statistic value (p-value) < 0.05. The significant values are marked in bold. The reverse causality (e.g., $S2 \rightarrow S1$) is mentioned under the *p*-value of each column ($S1 \rightarrow S2$)

To maintain a unique common nature across scouts for all subjects, I average the scout time series data across 26 subjects. Then, the statistical test (F-test) is performed on the average time series data of two scouts to find the direction of information flow between scouts (refer to Table 2 ) for both target and non-target stimuli. For 0-back, GC is observed from the visual to the imaginal scout and procedural to the imaginal scout. The causal flow between scouts is represented by the direction of GC. For the 2,3-back tasks, the imaginal module stores previous *n*-back letters in intermediate memory and send them to the retrieval module as per the demand of the procedural module. Thus, the GC occurs (in both directions) between the imaginal and the retrieval scout for 2-back and 3-back tasks. GC also occurs from the procedural to imaginal ACT-R module for all workload levels to control the tasks. The statistical results of information flow also validate the outcomes of GC analysis.

## Discussion and conclusion

The proposed framework highlights the information flow among ACT-R modules through different effective connectivity methods. The ACT-R modules are mapped to the respective brain scouts in the cortex surface. As per my knowledge, it is the first study that reveals the connectivity among ACT-R modules through brain cortices for the EEG-based cognitive task. The experiment is performed on an EEG-based *n*-back task with three workload levels (0, 2,  and 3-back). In ACT-R cognitive architecture, several modules (visual, aural, declarative memory, motor, working memory/imaginal, procedural, and goal) work together to generate the desired human behavior. Each module is activated while performing the designated task. In the ACT-R, Working memory (WM) is an essential part of elemental cognitive processing, as it works as a short-term memory that holds some intermediate result Glavan and Houpt (2019); Zhang et al. (2018). In the EEG-based *n*-back task, the working memory load increases with the increasing value of *n* Grissmann et al. (2017). Similarly, in 2-back and 3-back tasks, the working memory module (i.e., imaginal module) of ACT-R is more responsible for storing the intermediate result (previous *n* letters in the sequence). The intermediate result is retrieved later from the retrieval buffer for checking the target or non-target letter. Each ACT-R module is associated with some neural activities in a specific brain area. Therefore, it is necessary to identify the brain scouts/ROIs corresponding to ACT-R modules. Here, EEG source imaging is performed to find the respective ROI/scouts mapped with ACT-R modules and then validate those scouts with locations of Brodmann areas. Then, the activation time series for each brain scout is extracted, and the two effective connectivity methods are applied between different scouts/ACT-R modules. The changes in scout activation and brain connectivity analysis at the cortex surface for a specific time interval (1 s) highlight the activation of ACT-R modules and underlying brain dynamics between modules (Fig. 3).

It can be shown that retrieval, imaginal ACT-R modules are more activated at 3 s during the retrieval operation of the 2,3-back task, whereas the procedural module is activated most of the time (excluding the 2 and 4 s). In Figs. 5, 6, and 7, the information flow among ACT-R modules is demonstrated using GC and MTE along with the scout activation. A common observation is observed in the information flow for GC and MTE. For the 0-back task, information flow presents from the visual to imaginal modules for storing the stimulus in short-term memory. After stimulus presentation, in 2 and 3-back tasks, the information flow is observed from the imaginal to the retrieval module to store the current letter in declarative memory (DM) storage. Next, when the next letter appears, the callback operation is performed to retrieve the previous letter from DM, then information flow is observed between retrieval and imaginal module. Information flow is observed between procedural and imaginal modules to execute production for all tasks. The linear effective connectivity analysis through GC is performed between the activation time series of different scouts (Fig. 5a–l). In contrast, the non-linear effective connectivity through MTE is shown in Fig. 7. Two different effective connectivity methods are analyzed to find the relationship between the ACT-R module during the *n*-back task. As the GC refers to the ratio of variances of two-time series data, *F*-statistic is used to evaluate the statistical inference of GC analysis and identify the direction of information flow between scouts (Table 2).

In the near future, the proposed model will be implemented with other cognitive architecture (CLARION, Soar, LIDA, DUAL etc.) models to improve the effectiveness of the study.

## Footnotes

1 https://brmlab.cz/project/brain_hacking/broadmannarea
